# *Ptychadena* in Mauritania and the first record of *Ptychadena schillukorum*

**DOI:** 10.3897/zookeys.673.10265

**Published:** 2017-05-15

**Authors:** Alberto Sánchez-Vialas, Marta Calvo-Revuelta, Rafael Márquez

**Affiliations:** 1 Collection of Amphibians and Reptiles, Museo Nacional de Ciencias Naturales-CSIC, C/José Gutiérrez Abascal 2, 28006, Madrid, Spain; 2 Fonoteca Zoológica. Dept. de Biodiversidad y Biología Evolutiva, Museo Nacional de Ciencias Naturales-CSIC, C/José Gutiérrez Abascal 2, 28006, Madrid, Spain

**Keywords:** Herpetological collection, identification key, *Ptychadena schillukorum*, Sahel, taxonomic problems, West Africa

## Abstract

The study of specimens of the genus *Ptychadena* of the herpetological collection of the Museo Nacional de Ciencias Naturales de Madrid reveals the first record of *Ptychadena schillukorum* from Mauritania, extending the known distribution range of the species in West Africa more than 450 km northwards. A key is provided for the four Mauritanian species of *Ptychadena* to solve problems in identification encountered in previous studies.

## Introduction

*Ptychadena* Boulenger, 1917 is a genus of medium-sized Afrotropical frogs that comprises 50 known living species (Frost 2017) which occur mainly in savannahs and wetlands. Several species of *Ptychadena* share a similar appearance and show interspecific overlap in morphological variation hampering the identification of diagnostic characters (Poynton 1970, Rödel 2000). Padial et al. (2013) show a map of Mauritania with the known localities of all amphibian species represented except for *Ptychadena*, where the locations are pooled by genus, due to the ambiguity of its specific identification in literature. However, Guibé and Lamotte (1957) revised the *Ptychadena* of West Africa providing a morphological key and Bwong et al. (2009) and Dehling and Sinsch (2013) suggested that morphologically similar species of *Ptychadena* may be identified using quantitative and qualitative morphological data (Zimkus et al. 2017).

The western portions of the Sahel ecoregion includes a diversified group of species of *Ptychadena*, namely *P. pumilio* (Boulenger, 1920), *P. bibroni* (Hallowell, 1845), *P. tellini* (Peracca, 1904) (as *P. schubotzi* (Sternfeld, 1917)), *P. longirostris* (Peters, 1870), *P. oxyrhynchus* (Smith, 1849), *P. trinodis* (Boettger, 1881), *P. mascareniensis* (Dúmeril & Bibron, 1841), *P. tournieri* (Guibé & Lamotte, 1955), *P. stenocephala* (Boulenger, 1901), *P. submascareniensis* (Guibé & Lamotte, 1953), *P. retropunctata* (Angel, 1949), and *P. schillukorum* (Werner, 1908) (as *P. floweri*, (Boulenger 1917)) (Rödel 2000). At the northwestern limit of this area, along southern Mauritania, *Ptychadena* is represented by three species: *P. trinodis*, *P. mascareniensis* and *P. bibroni* (Padial 2003, Padial et al. 2013, Padial and De la Riva 2004). *Ptychadena mascareniensis* comprise a group of closely related species widely distributed across Africa but the taxonomy and phylogenetic affinities of the western populations from Senegal to Mauritania are still unresolved (Padial and De la Riva 2004, Vences et al. 2004, Zimkus 2017).

**Figure 1.**
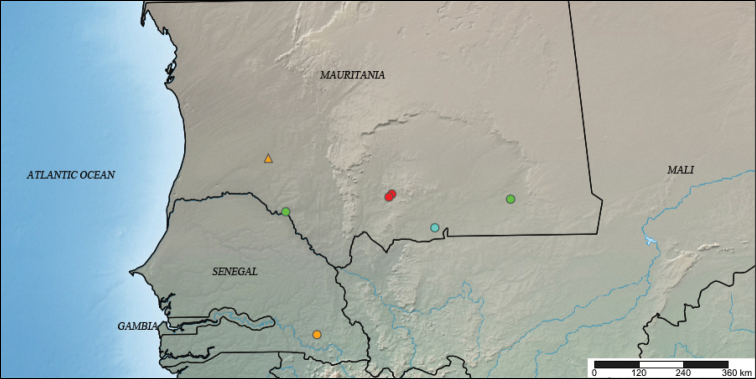
Geographic records published of the species of *Ptychadena* from Mauritania identified at species level. Yellow dot from Senegal represent the previous northernmost record in West Africa of *P. schillukorum*. Green dot represent the localities were *P. bibroni* has been recorded in Mauritania. Localities with presence of *P. trinodis* are represented by red dots. The record of P. cf. mascareniensis is the blue coloured dot. The new record of *P. schillukorum* from Mauritania is shown as a yellow triangle (map created from http://www.simplemappr.net/).

*Ptychadena trinodis* differs from the other two species of *Ptychadena* recorded in Mauritania by the presence of a tarsal tubercle and by very large inner and outer metatarsal tubercles; in Mauritania it has been reported in two localities: Guelta Oumm Lebare and Guelta Metraucha. Ptychadena cf. mascareniensis is cited only at one locality in Mauritania: Zoueina; it differs from *P. bibroni* by a very subtle outer metatarsal tubercle, lack of supernumerary tubercles on toe IV, dorsal median ridges that reach the posterior edge of the eyes, different webbing formula (2^2/3^–3 in P. cf. mascareniensis and 2–2^1/3^ in *P. bibroni*), and by a continuous lateral ridge which is broken in *P. bibroni. Ptychadena bibroni* is known by two records in Mauritania: Kaedi and Mahmoûdé Lake (Lamotte and Ohler 1997, Rödel 2000, Padial 2003, Padial and De la Riva 2004) (Fig. 1).

Abrupt climate changes and hydrological fluctuations that took place in southern Mauritania during Pleistocene–Holocene, created a dynamic transitional zone promoting dispersal and contraction of distributional areas of Afrotropical species (Gasse 2000; Foley et al. 2003; Brito et al. 2014; Sow et al. 2015). Most amphibians in Mauritania are restricted to the southern part of the country where they inhabit the Sahel savanna ecoregion (including all known *Ptychadena*). Only *Hoplobatrachus occipitalis*, *Sclerophrys xeros*, and *Tomopterna cryptotis*, are present across the north in the Saharan realm (Padial et al. 2013).

In this note, a new species record of *Ptychadena* is reported from southern Mauritania based on material of the herpetological collection of the Museo Nacional de Ciencias Naturales of Madrid (MNCN) and on field data obtained by RM.

## Material and methods

The specimens of *Ptychadena* held at the National Museum of Natural Sciences of Madrid (**MNCN-CSIC**) were examined. Thirty-one specimens were collected in Mauritania: one specimen of *Ptychadena trinodis* (cited in Padial 2003), one of P. cf. mascareniensis (cited in Padial and De la Riva 2004) and 29 individuals identified by us as *Ptychadena schillukorum*. Four specimens studied of *P. bibroni* were collected in Senegal (Table 1). Most individuals of *P. schillukorum* (19) were collected in October 1998 by RM, a few days after a (rare) event of rainfall in Southern Mauritania in the proximities of Aleg (“2 kilometers east of Aleg”, coordinates: 17°04'41"N, 13°53'41"W, and “west limit of Lake Aleg”, coordinates: 17°08'2"N, 14°04'0"W). Additionally, 10 individuals (MNCN 43846–43855) were collected in October 2006 by J. M. Padial and I. De la Riva in Aleg surroundings (17°30'10.0"N, 13°54'50.1"W) (Fig. 1).

**Table 1.** Morphological characters of MNCN voucher specimens used in this study for comparing between the species of *Ptychadena* present in Mauritania.

	*P. bibroni*	*P. cf. mascareniensis*	*P. trinodis*	*P. schillukorum*
Catalog number (MNCN)	44071–44074	41784	41783	43846–43855; 42221–42231; 42208; 42210–42216
Tarsal tubercle	absent	absent	present	absent
Inner metatarsal tubercle as long as toe I	absent	absent	present	absent
Outer metatarsal tubercle distinctive	present	absent	present	absent
Lateral ridge continuous	absent	?	present	absent
Dorsal ridges continuous	present	?	present	absent
Mottled pattern of thigh	absent	present	absent	present
Number of phalanges of toe 4 free of web	2	3	2	3
Supernumerary tubercles on toe IV	present	absent	absent	absent

Morphological characters used in this study for comparison between the species of *Ptychadena* from Mauritania are: tarsal tubercle, inner metatarsal tubercle, outer metatarsal tubercle, lateral ridge, dorsal ridge, thigh pattern, supernumerary tubercles on toes and webbing extension in the fourth toe (Table 1).

The measure of the range extension of *P. schillukorum* was taken by airline distance using Google Earth tool, from the northern most locality in West Africa at Niokolo-Koba in Senegal to Aleg in Mauritania.

## Results and discussion

The poor conditions in which we found the specimen of P. cf. mascareniensis collected in Mauritania by Padial and De la Riva (Padial and De la Riva 2004) makes its identification difficult due to the lack of morphological characters such as the dorsal and lateral ridges. The existence of three phalanges of the fourth toe free of web and the mottled pattern of thigh makes us question its identity (see Table 1). Unfortunately, the only existing picture provided by J. M. Padial (Fig. 5C) of the same specimen does not allow us to conclude its specific identification as *Ptychadena* of the *mascareniensis* group. Genetic samples of it will be analysed to solve this question (in prep.). In this work the criteria of Padial and De la Riva considering the presence of P. cf. mascareniensis in Mauritania are followed.

The morphology of the studied individuals of *Ptychadena schillukorum* from Mauritania fitted with the description of the species (Channing and Howell 2006, Rödel 2000): dorsal and lateral ridges are not continuous; the legs are relatively short: tibiotarsal articulation reaches the level of the eye; lack of tarsal tubercles; outer metatarsal tubercles are inconspicuous; the pattern of thigh are speckled (fine vermiculation); the webbing is reduced: 2.5-three phalanges of the fourth toe free of web and no supernumerary tubercles are present in the toes. Tympanum small: its diameter reaches less than 0.7 diameter of the eye.

The examined individuals MNCN 42223, MNCN 42224, MNCN 42226, MNCN 42229, MNCN 42212, MNCN 42215, MNCN 42216 and MNCN 43849, present a thin yellow vertebral stripe. Therefore, the examined specimens are characterised by the tibia pattern with interrupted transversal bands.

The nearest locality to Mauritania where *Ptychadena schillukorum* has been reported is in southern Senegal at Niokolo-Koba (Lamotte 1969, Joger and Lambert 2002). The record from Aleg in Mauritania extends 450 km northwards the distribution of the species (Fig. 1).

**Figure 2.**
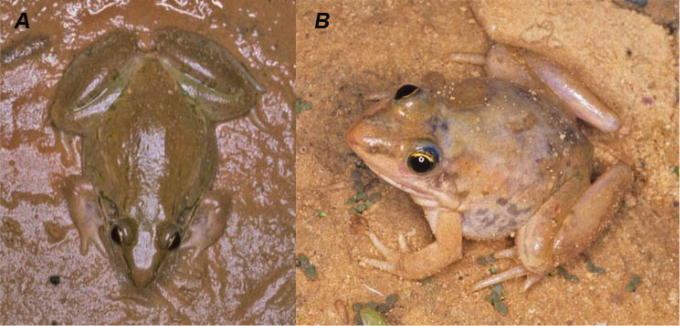
Different individuals of *Ptychadena schillukorum* from Aleg, Mauritania. Museum number of photographed specimens: **A** MNCN 42210 (SVL = 32.7 mm) **B** MNCN 42221 (SVL = 27.3 mm) (photos Rafael Márquez).

*Ptychadena schillukorum* (Fig. 2A, B) inhabits the dry savannahs and grasslands from Senegal to the extreme southern Somalia and also in isolation from Egypt (Baha el Din 2006, Nago et al. 2006) and southwards to east Africa from Kenya to Mozambique (Channing and Howell 2006). The reproduction of *P. schillukorum* in West Africa takes place in shallow savannah ponds during the rainy season (Nago et al. 2009).

**Figure 3.**
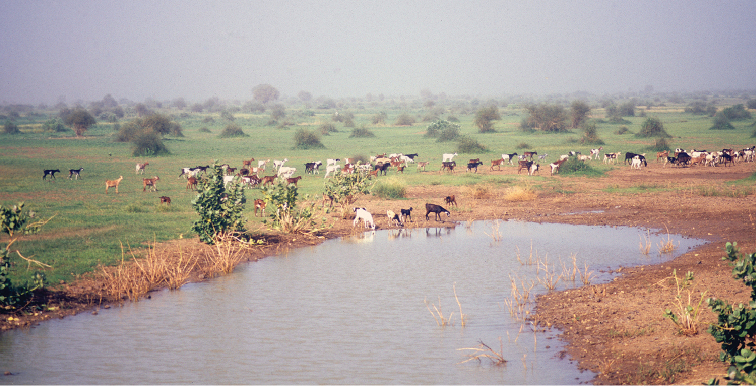
Temporal pond where *Ptychadena schillukorum* was found in Aleg, Mauritania (photo Rafael Márquez).

**Figure 4.**
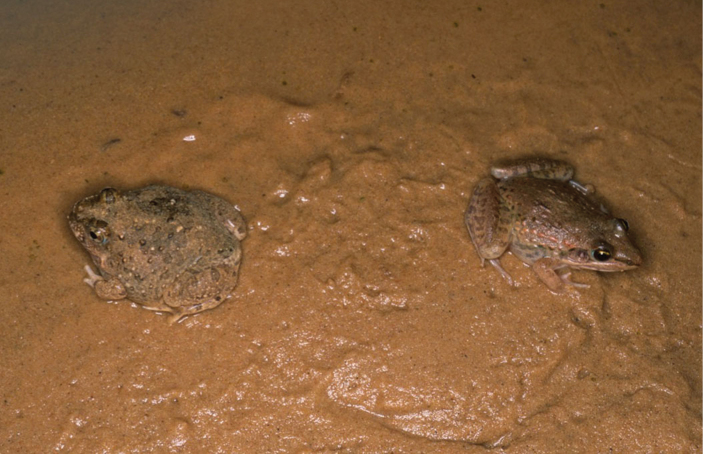
*Tomopterna cryptotis* and *Ptychadena schillukorum* (museum number: MNCN 42214, SVL = 31.8 mm) found in syntopy, Aleg, Mauritania (photo Rafael Márquez).

Aleg is located in the Sahel region, characterised by a subtropical dry arid climate with rainy season from July to October. Most individuals were collected in October 1998 by RM, a few days after a (sporadic) event of rainfall that caused the formation of shallow pools and the growth of herbaceous vegetation on the normally barren sand (Fig. 3). Other anuran species that were found concomitantly were *Sclerophrys xeros* and *Tomopterna cryptotis* (Fig. 4).

**Figure 5.**
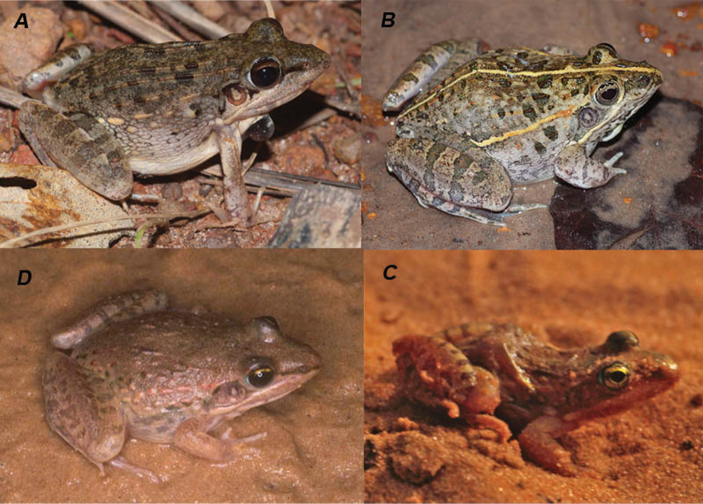
The four species of *Ptychadena* recorded in Mauritania: *Ptychadena bibroni* from Gourel Yoba, Senegal; male specimen, not collected (**A**), *Ptychadena trinodis* from Toubakouta, Senegal; male specimen, not collected (**B**), Ptychadena cf. mascareniensis from Zoueina, Mauritania (MNCN 41784, SVL=29.6 mm) (**C**), *Ptychadena schillukorum* from Aleg, Mauritania (MNCN 42214, SVL=31.8 mm) (**D**) [photos Alberto Sánchez Vialas (**A, B**), José Manuel Padial (**C**) and Rafael Márquez (**D**)].

The four Mauritanian species of *Ptychadena* (Figure 5) are easily distinguishable from each other based on morphological characters. *Ptychadena schillukorum* differs from *P. trinodis* by the lack of tarsal tubercle, smaller inner metatarsal tubercle and inconspicuous outer metatarsal tubercle. Also a fine yellow vertebral line is sometimes present (8 of 29 specimens of *P. schillukorum* examined) while in *P. trinodis* a fine white-yellow vertebral line and light colored lateral ridge is generally present. *Ptychadena schillukorum* differs from *P. bibroni* by its more compact body, shorter legs, lack of supernumerary tubercles on toe IV, always interrupted dorsal ridges, by its inconspicuous outer metatarsal tubercle and by thigh pattern, that is speckled in *P. schillukorum* and usually with yellow longitudinal and irregular line in *P. bibroni*. Finally, *P. schillukorum* is distinctive from *P. mascareniensis* by its compact body shape, dorsal and lateral ridges, always interrupted, and shorter legs.

Based on Rödel (2000) and personal data, a key for the *Ptychadena* species that occur in Mauritania is presented.

### Key to the species of *Ptychadena* from Mauritania

**Table:** 

1	Tarsal tubercle present. Metatarsal tubercles large. Inner metatarsal tubercle almost as long as the toe 1	***P. trinodis***
–	Lack of tarsal tubercle. Inner metatarsal tubercle smaller than 0.6 of the length of toe 1	**2**
2	Dorsal ridges continuous	**3**
–	Dorsal ridges discontinuous, broken into warts.	***P. schillukorum***
3	Dorsal median ridges to mid-dorsum. Lateral ridge discontinuous. Supernumerary tubercles on toe IV present	***P. bibroni***
–	Dorsal median ridges to posterior edge of the eyes. Lateral ridge continuous. Supernumerary tubercles on toe IV absent	***P. mascareniensis***

Studies concerning amphibian fauna in Mauritania are scarce, suggesting that other populations of *Ptychadena schillukorum* may be discovered in the future. Molecular studies of *P. schillukorum* should be carried out to know how complex is the species across their distribution (expected to be high or a group of species as suggested by Nago et al. 2006) and also to determine what is the systematic position and taxonomy of the Mauritanian population.
